# Therapeutic Approach to the Management of Severe Asymptomatic Hyponatremia

**DOI:** 10.1155/2017/1371804

**Published:** 2017-07-27

**Authors:** Thaofiq Ijaiya, Sandhya Manohar, Kameswari Lakshmi

**Affiliations:** Department of Medicine, Montefiore New Rochelle Hospital, Albert Einstein College of Medicine, New Rochelle, NY, USA

## Abstract

Hyponatremia is an electrolyte imbalance encountered commonly in the hospital and ambulatory settings. It can be seen in isolation or present as a complication of other medical conditions. It is therefore a challenge to determine the appropriate therapeutic intervention. An understanding of the etiology is key in instituting the right treatment. Clinicians must not be too hasty to correct a random laboratory value without first understanding the physiologic principle. We present such a case of a patient who presented with sodium of 98 mmol/L, the lowest recorded in the current literature, and yet was asymptomatic. Following appropriate management driven by an understanding of the underlying pathophysiologic mechanism, the patient was managed to full recovery without any clinically significant neurological sequelae.

## 1. Introduction

Hyponatremia is the most commonly identified electrolyte abnormality in hospitalized adults [1, 2] and known to have an association with mortality. Symptoms can range from nausea and malaise, with mild reduction in the serum sodium, to lethargy, decreased level of consciousness, and, in severe cases, seizures and coma [3, 4]. Overt neurologic symptoms are due to very low serum sodium levels (usually less than 115 mEq/L), resulting in intracerebral osmotic fluid shifts and brain edema. Cases of severe hyponatremia presenting with no neurologic symptoms are rare [5]. We report a case of severe, asymptomatic hyponatremia with a sodium level of 98 mmol/L, which to the best of our knowledge is the lowest recorded level in the current literature.

## 2. Case Report

A 48-year-old female presented to our emergency room (ER) with facial injuries following a mechanical fall. She denied any dizziness, chest pain, palpitations, or unsteadiness of her gait prior to this.

Her history was remarkable for alcoholic liver disease with prior episodes of nonvariceal gastrointestinal bleeding. She continues to consume alcohol on a regular basis, usually 2 bottles of beer per day and her last drink was a day prior to her fall. She also smokes up to a pack of cigarettes a day. Her home medications were potassium supplement and multivitamins.

On physical examination, she had a pulse rate of 78/min and a blood pressure of 154/59 mmHg with no orthostatic changes. She appeared comfortable and well oriented to time, place, and person. She had moist mucous membrane and no JVD. She had multiple lacerations on her face that were sutured in the ER, along with multiple bilateral ecchymotic patches on her legs. Her neurological exam showed mild impairment in her balance and coordination. Rest of the physical examination was unremarkable.

Blood work showed serum sodium concentration was 98 mmol/L, and other laboratory data are listed in Table 1. Her baseline serum sodium from a year ago was consistently between 125 and 129 mmol/L with no symptoms reported and there was no intervention at that time. Her stools were positive for occult blood. A chest X-ray and a CT head were normal.

A decision was made to admit the patient to the Intensive Care Unit for closer monitoring of her sodium levels. Her volume status was considered to be low initially due to presumed gastrointestinal bleed as well as the low urine sodium and high urine osmolality. A volume challenge with one litre of 0.9% saline was initially given. A repeat serum sodium level obtained 2 hours later was 100 mmol/L. She was subsequently transfused with 2 units of packed red cells. However, urine osmolality remained elevated (337 mOsm/kg) following this with no significant change in serum sodium levels suggesting an underlying persistent high ADH state. Absence of typical symptoms of severe hyponatremia suggested a chronic etiology as did her moderate hyponatremia from a year earlier.

**Figure 1:**
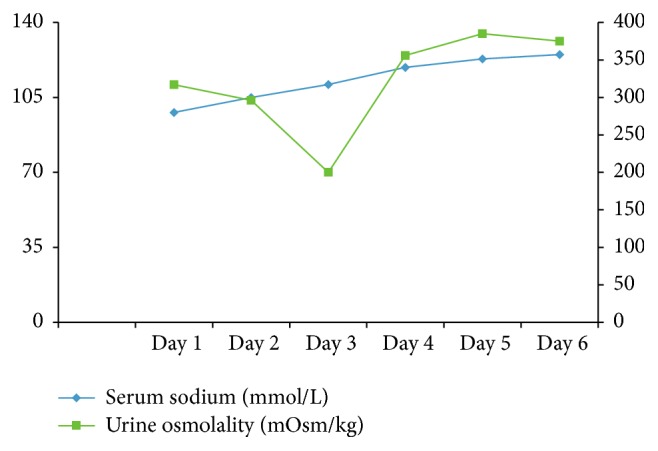
Daily trend of serum sodium plotted on left *y*-axis and urine osmolality plotted on right *y*-axis.

She was placed on fluid restriction of up to 800 ml/day. Her hypokalemia and hypomagnesemia were corrected with infusions of potassium chloride and magnesium sulphate. With this approach, her serum sodium increased by 7 mmol in the first 24 hours (Figure 1). She had a gradual improvement in her sodium concentration with her levels reaching 125 mmol/L over a six-day period. She had a CT of the chest, abdomen, and pelvis and it did not reveal any significant findings. Her liver dysfunction was managed supportively during her hospital course. The patient was subsequently discharged without any neurological sequelae and follow-up planned with nephrology as well as hepatology.

## 3. Discussion

Hyponatremia is the clinical manifestation of a wide variety of diseases and identifying the underlying mechanism is crucial in instituting the right treatment [6, 7]. An inappropriate treatment may cause more harm than the initial presenting condition; thus, clinicians need to be familiar with the diagnosis and management of various forms of hyponatremia. The determination of the underlying pathophysiologic mechanism requires a detailed history with emphasis on medications and social habits, a thorough physical exam to assess the volume status, and the valuable input of laboratory and radiological data.

Serum sodium is one of the main determinants of serum osmolality. A fall in serum sodium concentration results in the development of cerebral edema from the sudden changes in osmolality. The brain's adaptation to the process begins immediately after the initial fall in serum osmolality and is completed in 2-3 days [8]. The brain adapts by losing its organic solutes with a resultant osmotic movement of water out of the cell, thus reducing brain swelling [9]. The hyponatremia in such a patient where the brain has acclimatized to a new homeostasis is considered to be chronic hyponatremia [8]. When the hyponatremia is corrected, this process of adaptation in the brain reverses. An aggressive reversal of chronic hyponatremia does not give the brain sufficient time for reuptake of the organic solutes and water resulting in cell shrinkage and demyelination. This is known as osmotic demyelination [10]. It can lead to irreversible neurologic dysfunction, seizures, coma, and, in severe cases, death.

The determination of chronicity is a challenging clinical scenario. It is suggested to consider the patient's history, prior baseline laboratory data as well as the neurological clinical picture to determine chronicity. It is important for the clinician to be aware of this challenge as impulsive decisions can lead to deleterious effects during treatment [11]. Our patient presented with profoundly low serum sodium concentration which appears to have been chronic in nature considering her lack of neurological symptomatology. When in doubt, it is safer to presume the condition to be chronic and be cautious in the management.

Although the existence of truly asymptomatic hyponatremia has been questioned [12, 13], our patient did not manifest with any clinically significant neurological symptoms like seizures or confusion. It could be argued that her mild gait imbalance was related to her hyponatremia although our patient did not provide a history to make that association. The gait abnormality described in this case may be due to other neurological dysfunctions (e.g., cortical atrophy, cerebellar dysfunction) stemming from her chronic alcohol use. Her neurological exam also did not change despite the correction of her sodium. Renneboog et al. have described in their study an association between mild chronic hyponatremia with an increasing incidence of gait and attention impairments [13]. But the general consensus supports the evidence that clinical presentation of severe hyponatremia is influenced more by the rate of decline of the sodium level as against the absolute value [14, 15].

The hyponatremia in our patient was determined to be multifactorial and attributable to a combination of hypovolemic hyponatremia superimposed on chronic hyponatremia related to syndrome of inappropriate antidiuretic hormone secretion (SIADH). The low potassium store, which was attributed to her poor nutrition status from alcoholism, was also likely a propagating factor. Potassium is an osmotically active solute like sodium and the repletion of low potassium levels will increase the serum osmolality and result in the shifting of sodium from intracellular to extracellular space [16]. Monitoring and provision of other osmotically active substrates such as serum phosphate and magnesium are also necessary in the correction of hyponatremia by reducing osmolality differences between compartments [17]. Our patient was managed with the guiding principle that the serum sodium concentration should be corrected at a rate of no more than 10 meq/L in the first 24 hours, 18 meq/L in the first 48 hours, and 20 meq/L in the first 72 hours to prevent iatrogenic brain injury and central pontine myelinolysis [18, 19]. It is recommended that, in high-risk patient groups (severe malnutrition, alcoholism, or advanced liver disease), therapeutic target range should be below the limits that have been established for patients without these conditions as they are at higher risk of osmotic demyelination [19]. Hypertonic saline is not recommended in the management of asymptomatic hyponatremia [18].

Our patient humbled us with a perilously low serum sodium level of 98 mmol/L which to the best of our knowledge is one of the lowest serum sodium concentration reported in the current literature. Joseph et al. discuss a patient with serum sodium of 99 mmol/L who improved on fluid restriction without any neurological sequelae [20]. Accurate diagnosis of the etiology by understanding the underlying mechanism at play is key to a successful correction. Severe hyponatremia, even with such critically low sodium concentration, with appropriate management and diligent monitoring can be managed to the extent of full recovery without any clinically significant neurological sequelae.

## Figures and Tables

**Table 1:** Laboratory data at the time of admission.

Sodium 98 mmol/L
Potassium 2.6 mmol/L
Magnesium 1.1 mmol/L
Blood urea nitrogen 19 mg/dL
Creatinine 0.71 mg/dL
Creatine kinase 2366 IU/L
Measured osmolality 235 mOsm/kg
Calculated osmolarity 208 mOsm/kg
Glucose 97 mg/dL
Cortisol 43mcg/dL
TSH 1.55 mIU/L
Total bilirubin 1.8 mg/dL
INR 1.5
Hemoglobin 6.0 mg/dL
Mean corpuscular vol. 93
White blood cell 8,000/mcL
Platelet 67,000/mcL
Folate 9.1 ng/mL
Vitamin B12 1500 pg/mL
Blood alcohol level 82 mg/dL
Urine osmolality 317 mOsm/kg
Urine sodium 17 mmol/L
Urine potassium 31 mmol/L
Urine chloride 25 mmol/L
Direct bilirubin 0.6 mg/dL
AST/ALT 404/113 IU/L
